# Feasibility of a Virtual Mindfulness-Based Stress Reduction Intervention for Individuals With Chronic Pain: Pilot Randomized Controlled Trial

**DOI:** 10.2196/98632

**Published:** 2026-09-23

**Authors:** Shashank Ghai, Sabrina Cavallo, Mathieu-Joël Gervais, Diana Zidarov

**Affiliations:** 1 Department of Political, Historical, Religious and Cultural Studies, Karlstad University, Karlstad, Värmland, Sweden; 2 Centre for Societal Risk Research, Karlstad University, Universitetsgatan 2, Karlstad, Värmland, 65188, Sweden, 46 0702904119; 3 School of Rehabilitation, Faculty of Medicine, Université de Montréal, Montréal, QC, Canada; 4 Institut universitaire sur la réadaptation en déficience physique de Montréal (IURDPM), Centre intégré universitaire de santé et de services sociaux du Centre-Sud-de-l'Île-de-Montréal, Montréal, QC, Canada; 5 Centre for Interdisciplinary Research in Rehabilitation of Greater Montreal (CRIR), Montréal, QC, Canada; 6 Centre de recherche Azrieli du centre hospitalier universitaire Sainte-Justine (CRCHUSJ), Montréal, QC, Canada; 7 Department of Psychology, Université de Montréal, Montréal, QC, Canada

**Keywords:** mindfulness, pilot study, feasibility, pain reduction, stress, randomized

## Abstract

**Background:**

Chronic pain (CP) is a complex and prevalent public health condition that significantly impacts physical functioning, psychological well-being, and quality of life. Traditional biomedical treatments often provide limited relief, highlighting the need for integrative approaches. Biopsychosocial interventions, such as mindfulness-based stress reduction (MBSR), have shown promise in improving pain-related outcomes by targeting cognitive, emotional, and behavioral processes associated with CP.

**Objective:**

This pilot randomized controlled trial (RCT) evaluated the feasibility and preliminary effects of a virtual MBSR intervention for individuals with CP.

**Methods:**

We conducted a single-blinded, randomized, pilot trial of 61 participants with CP randomly assigned to either an 8-week virtual MBSR program or a no-intervention control group. Participants were aware of their allocation; the data analyst remained blinded throughout the study; and the outcome assessor was blinded at baseline, although blinding could not be reliably maintained at subsequent assessments. The primary outcome was feasibility, assessed through recruitment, retention, and adherence rates. Secondary outcomes, examined on an exploratory basis, included pain disability, CP acceptance, pain catastrophizing, pain self-efficacy, health-related quality of life (HRQoL; PROMIS-29 [Patient-Reported Outcomes Measurement Information System-29] and PROMIS-10 [Patient-Reported Outcomes Measurement Information System-10]), and global rating of change.

**Results:**

Recruitment (23.5%), retention (60.7%), and adherence (63.3%) rates were below the prespecified thresholds of 30%, 80%, and 80%, respectively, indicating that modifications to the current design are required before proceeding to a definitive trial. As this pilot trial was not powered to test intervention effectiveness, the following clinical outcomes are reported as exploratory and hypothesis-generating. A significant group-by-assessment period interaction was observed for pain disability, PROMIS-10 (physical health and global health), and PROMIS-29 (participation in social activities). No significant interactions were observed for pain catastrophizing, pain self-efficacy, or CP acceptance. On the Global Rating of Change scale, MBSR participants reported greater improvement in pain self-efficacy than the no-intervention control group.

**Conclusions:**

This pilot study did not meet its feasibility targets, highlighting the need to refine recruitment, retention, and intervention delivery procedures before conducting a future trial. Preliminary exploratory analyses suggested improvements in pain disability and several HRQoL subdomains in the MBSR group compared with the control group, but these findings should be interpreted cautiously given the underpowered sample and the exploratory nature of these analyses. These results provide guidance for optimizing study design and implementation of a future, adequately powered RCT evaluating virtual MBSR for adults with CP.

## Introduction

Chronic pain (CP) is a widespread condition affecting a substantial proportion of the population, and its management is recognized as a fundamental human right [1]. It is defined as pain persisting in 1 or more body regions for more than 3 months, beyond the expected period of tissue healing [1,2]. Unlike acute pain, which serves a protective function, CP may persist after its warning role has ended and no longer provide a clear biological benefit [1,3]. Its persistence is associated with alterations in both peripheral and central nervous system processes [4]. Despite its burden, several aspects of CP remain inadequately addressed. Public awareness of CP and its socioeconomic consequences remain limited, while many individuals experience barriers to timely and appropriate care. Furthermore, health care professionals often report insufficient training and confidence in CP management, and access to specialized pain services remains limited in many regions [4].

In Canada, approximately 19% of the population have been reported to experience CP, with roughly two-thirds experiencing moderate to severe pain intensity [1,5,6]. This high prevalence has far-reaching consequences, including a reduced quality of life, declines in physical health, and difficulties maintaining interpersonal relationships. Individuals with CP also frequently report sleep disturbances, employment challenges [7,8], and higher rates of depression [2,4].

Despite its significant impact on physical, psychological, and social aspects of health-related quality of life (HRQoL), there are currently no curative treatments available to effectively manage CP [9,10]. The current evidence base indicates that the most effective way to address this condition is through patient-centered, multidisciplinary, biopsychosocial care that supports individuals in coping with and managing daily activities while living with CP [2,11]. Although multidisciplinary and specialized pain clinics have demonstrated effectiveness in the management of CP [12,13], the rising prevalence of CP has increased demand and created challenges in accessing these comprehensive services.

In 2017‐2018, the average wait time to access these multidisciplinary services in Canada was approximately 7.9 (SD 10.9) months [14]. These prolonged waiting periods, which far exceed the recommended 2-month time frame set by the International Association for the Study of Pain, lead to a deterioration in the condition of individuals with CP [15]. This deterioration manifests as increased health care costs and a decreased overall quality of life [16,17]. Given the uncertainty regarding when or whether these waiting times will decrease, particularly as the population grows and ages, it is crucial to develop interventions that address not only the intensity of pain, but also how it affects an individual’s daily functioning [5].

One promising approach in this regard is mindfulness-based stress reduction (MBSR) [18,19]. MBSR draws on the concept of mindfulness meditation, originally rooted in the Buddhist spiritual tradition, and integrates it into behavioral treatment approaches for individuals with CP [20,21]. Mindfulness involves intentionally directing attention to the present moment, including one’s cognitions, perceptions, and emotions, without judgment or any intent to control or alter them [22,23]. MBSR trains individuals in mindfulness practices to decrease cognitive vulnerability to stress and emotional distress. The program also helps people distinguish the sensory aspect of pain from its cognitive and emotional components, which may be why it is effective at reducing pain intensity [23]. Neuroimaging studies offer a plausible mechanism for MBSR’s effects on pain, indicating that it may modulate pain perception by altering its contextual appraisal, a process influenced by factors such as attention, emotion, prior experiences, expectations, and the perceived meaning of pain [24]. Therefore, it can be inferred that MBSR targets the psychological and cognitive correlates of pain, including attention regulation, emotional reactivity, cognitive appraisal, and catastrophizing, with the goal of enhancing overall functioning, body awareness, pain acceptance, and tolerance of discomfort, while reducing distress rather than exclusively focusing on pain elimination [23]. Importantly, evidence suggests that MBSR can also be delivered virtually, with outcomes comparable with in-person training, offering a potentially cost-effective and accessible option for participants who might otherwise face barriers [25]. As a result, virtual MBSR could be a valuable complementary approach for individuals on long waiting lists, providing interim support while they await access to specialized pain services.

Recent systematic reviews evaluating MBSR’s impact on CP have found mixed results. Most reviews indicate small-to-moderate effects [22,26-32], or no effects [33-35], of MBSR in improving pain-related outcomes. These limited effects may be partly explained by the use of outcome measures that do not align with the intended goals of MBSR; for example, pain intensity is often assessed as a primary outcome, despite MBSR being designed to improve pain-related disability, coping, and self-management [27,30,36]. The reviews also highlight several methodological limitations in the existing literature, including poor trial quality, lack of follow-up, absence of intention-to-treat (ITT) analyses, inadequate randomization procedures, and substantial heterogeneity in outcome reporting [19,22]. Additionally, nonstandardized outcome reporting, inadequate mindfulness definitions, and incomplete intervention descriptions have limited understanding of MBSR’s true impact, highlighting the need for more rigorous research methodologies [19]. Beyond these methodological limitations, further research is needed to determine how MBSR can be effectively implemented in accessible formats and within specific health care contexts.

In particular, evidence regarding the delivery of virtual MBSR for CP remains limited, especially within the Canadian health care context and the province of Quebec. In Quebec, access to MBSR is limited, as programs are often offered through private services, which may be costly and therefore inaccessible to many individuals living with CP. Expanding the availability of MBSR within the public health care system could help reduce barriers to participation and support more equitable access to mind-body approaches to pain management. However, integrating a new intervention into existing health services requires thoughtful planning and evaluation [37]. Pilot studies are therefore essential to assess feasibility, refine procedures, identify implementation barriers and facilitators, and ensure appropriate methods before conducting a larger clinical trial.

The primary aim of this pilot trial was to evaluate the feasibility of delivering a virtual MBSR intervention for individuals with CP in Quebec, including recruitment, retention, and adherence. As a secondary exploratory objective, clinical outcomes were assessed to identify preliminary trends and inform the design of a future definitive trial, including outcome selection and sample size estimation. Outcomes included pain-related disability, pain self-efficacy, pain acceptance, pain catastrophizing, global perceived change in pain self-efficacy, and HRQoL.

## Methods

### Design

This pilot randomized controlled trial (RCT), conducted from May 2021 to February 2022, evaluated the feasibility and preliminary effects of MBSR compared with a control group across 3 assessment periods: preintervention (T1), postintervention (T2, 8 weeks after starting the intervention), and 3 months follow-up (T3).

### Ethical Considerations

This study was approved by the research ethics committee of the Center for Interdisciplinary Research in Rehabilitation of Greater Montréal (IRB CRIR-1499‐1120/multi). All participants signed informed consent, which included the therapist’s contact information for questions or concerns throughout the study. No compensation was provided for this study. A CONSORT (Consolidated Standards of Reporting Trials) checklist for pilot and feasibility trials [38] is provided in Checklist 1. The trial was preregistered at clinicaltrials.gov (NCT04842097) prior to enrollment.

### Participants and Recruitment

Individuals with CP on the waiting list for pain clinic services at the Constance-Lethbridge Rehabilitation Center and Hôpital de Verdun (CH Verdun) were approached by clinical research coordinators at each site to gauge interest. Individuals with CP who were members of the Association Québécoise de Douleur Chronique (AQDC) were also invited via a post on the association’s website. Interested individuals were contacted by a research assistant who explained the study and administered an eligibility questionnaire via REDCap. Eligibility criteria were as follows: (1) age 18 years or older; (2) chronic noncancer, nonmigraine pain of at least 3 months’ duration occurring at least 4 days per week; (3) pain intensity of 4 or higher on an 11-point scale (from 0 “no pain” to 10 “the most intense pain imaginable”); (4) ability to read and write in French or English; (5) ability to commit to a weekly 2-hour virtual MBSR session for 8 consecutive weeks; (6) no current or past-24-month coordinated care from a pain clinic, hospital, or rehabilitation center; (7) no more than 12 cognitive behavioral therapy sessions in the past year; and (8) internet access with the skills to use a laptop or tablet.

Individuals were excluded for an unstable psychiatric condition (including depression, bipolar disorder, posttraumatic stress disorder, and psychotic disorders with hallucinations or delusions) or current participation in a structured mindfulness or meditation program.

### MBSR

An experienced therapist trained in MBSR delivered the intervention via Zoom (Zoom Communications), following the core format and content of the standard 8-week MBSR program described by Kabat-Zinn [39]. The intervention involved 8 group sessions delivered weekly, lasting approximately 120 minutes each. The 30 participants allocated to the MBSR intervention were divided into 2 parallel groups of 15 to facilitate virtual group delivery and participant interaction. Sessions were held weekly in the afternoon, on the same day and at the same time for each group, scheduled according to participants’ availability during recruitment and kept consistent throughout the intervention.

Each session began with a 10-minute guided meditation, followed by a comprehensive 30-minute exploration of mindfulness concepts. This was succeeded by two 10-minute exercises, interspersed with a 10-minute break for reflection and rejuvenation. A 20-minute session further elaborated on mindfulness concepts, leading into a 30-minute period of group sharing, discussion, and further engagement with the material. Each session concluded with a 10-minute guided meditation. Participants attended the virtual sessions using their own devices equipped with cameras and microphones. The therapist encouraged participants to keep their cameras on to foster group presence and allow real-time monitoring of engagement and well-being, although camera use was not mandatory. Detailed session content is provided Multimedia Appendix 1.

During the first session, the therapist provided an overview of the program and its objectives. Participants were encouraged to practice mindfulness independently for at least 30 minutes, 6 days per week [40]. To support home practice, they received 2 recorded guided meditation sessions via email. Participants also maintained a daily journal documenting practice duration, emotional experiences, noteworthy observations, and any adverse events. They were encouraged to contact the therapist by email at any time to report adverse events or other concerns between sessions.

Following completion of the 8-week MBSR program, participants requested additional support to facilitate the transition to independent practice. In consultation with the therapist, 2 optional 45-minute group follow-up sessions were offered approximately 3 and 8 weeks after the intervention, focused on discussing home practice, identifying barriers and facilitators, and problem-solving strategies for integrating mindfulness into daily life. No new mindfulness content or intervention components were introduced. Attendance was not systematically monitored. Available records from the first cohort indicate that 8 participants attended the first session and 5 attended the second, although comparable data were not available for the second cohort.

### Control Group

Control participants did not undergo any intervention but completed the same questionnaires and assessments, at the same intervals, as the MBSR group.

### Randomization

The randomization sequence was generated using Microsoft Excel (version 16.18) with a 1:1 allocation ratio and blocks of 2 by a digital health engineer with no clinical involvement in the trial. The resulting allocation list was provided to a research assistant responsible for enrolling eligible participants and implementing the pregenerated randomization sequence.

### Blinding

This study used a single-blind design in which blinding of participants was not feasible due to the nature of the MBSR intervention. Outcome data were collected directly from participants through self-reported questionnaires administered via REDCap. The outcome assessor’s role was limited to sending follow-up reminders and had no role in questionnaire administration or data entry. The data analyst remained blinded throughout the study. Assessor blinding to group allocation was maintained at baseline (T1) but could not be maintained at subsequent assessments (T2 and T3), as follow-up interactions with participants may have revealed group allocation. A formal assessment of assessor blinding was not conducted.

### Assessments

#### Overview

Sociodemographic data included age, sex, education, marital status, occupation, pain duration, primary cause of CP, site or sites of CP, and current or previous pharmacological and nonpharmacological treatments. The outcome measures were selected in accordance with the latest guidelines in CP management [41,42], our own previous research [43], and recommendations from existing systematic reviews [34,40,44]. Given the exploratory nature of this pilot study, intervention effects were assessed across multiple health outcomes.

#### Primary Outcome Measures

The feasibility of the MBSR intervention was evaluated using 4 indicators: recruitment rate (percentage of individuals who agreed to participate among those contacted), adherence rate (percentage attending at least 4 of 8 MBSR sessions and completing at least half of the required home practice), retention rate (percentage reporting outcome measures at all 3 assessment periods), and average questionnaire administration time. Feasibility objectives were a minimum recruitment rate of 30%, retention rate of 80%, and intervention adherence rate of 80%, consistent with established methodological recommendations for pilot studies [45].

#### Secondary Outcomes Measures

First, the Pain Disability Index (PDI) is a 7-item instrument that assesses pain-related disability across family and home responsibilities, recreational activities, social interactions, work, sexual behavior, self-care, and basic life support activities [46]. Each item is rated from 0 to 10, yielding a total score of 0‐70, with higher scores indicating greater pain-related disability. The scale is reliable and valid [47].

Second, the Pain Catastrophizing Scale (PCS) measures catastrophic thinking about pain, asking individuals to recall past pain episodes and rate related thoughts and emotions [48]. Its 13 items assess rumination, magnification, and helplessness, with total scores from 0 to 52, higher scores indicating greater catastrophizing [49]. The PCS is a reliable and valid instrument [50].

Third, the Chronic Pain Acceptance Questionnaire (CPAQ) measures acceptance of CP, defined as experiencing ongoing pain without attempting to avoid, control, or reduce it. Its 8 items assess activity engagement and pain willingness [51], each rated 0 to 6, with total scores from 0 to 48, higher scores indicating greater acceptance. The CPAQ is a reliable and valid instrument [52].

Fourth, the Pain Self-Efficacy Questionnaire (PSEQ) measures confidence in performing daily activities while experiencing CP [53], with 10 items rated 0 (not at all confident) to 6 (completely confident), yielding total scores ranging from 0 to 60, higher scores indicating greater self-efficacy. The PSEQ is a reliable and valid instrument [54].

Fifth, the PROMIS-29 (Patient-Reported Outcomes Measurement Information System-29) is a 29-item survey evaluating physical function, fatigue, depressive symptoms, anxiety, pain interference, sleep disturbance, and participation in social roles and activities. Each domain is rated on a 5-point scale and converted to standardized T-scores, with 50 representing the general population average. The PROMIS-29 is a reliable and valid instrument [55].

Sixth, the PROMIS-10 (Patient-Reported Outcomes Measurement Information System-10) survey assesses self-reported physical, mental, global health, and social activities and role using a 5-point scale and has demonstrated reliability, precision, and comparability to prior assessment tools [56]. Physical and mental health scores are converted to standardized T-scores, with 50 representing the general population average.

Finally, the Global Rating of Change Scale (GRC) assessed participants’ perceptions of their self-efficacy in managing their pain compared with before the intervention. Participants responded using an 11-point scale ranging from −5 (“much worse”) to +5 (“very much improved”), with 0 indicating no change [57].

### Data Collection Procedure

The data collection schedule is presented in Table 1. Assessments were conducted using REDCap, a secure research data platform [58], across the 3 assessment periods.

**Table 1.** Data collection schedule for the pilot randomized controlled trial evaluating an 8-week virtual mindfulness-based stress reduction (MBSR) intervention versus no-intervention control condition among adults with chronic pain in Quebec, Canada (May 2021-February 2022). Assessments were conducted at baseline (T1), postintervention (T2; 8 weeks), and at 3-month follow-up (T3).

Outcome	T1	T2	T3
Primary outcomes			
Participation rate	✓		
Retention rate	✓	✓	✓
Adherence rate		✓	
Time to administer questionnaires	✓	✓	✓
Secondary outcomes			
PDI^a^	✓	✓	✓
CPAQ^b^	✓	✓	✓
PCS^c^	✓	✓	✓
PSEQ^d^	✓	✓	✓
PROMIS-29^e^	✓	✓	✓
PROMIS-10^f^	✓	✓	✓
GRC^g^		✓	

a PDI: Pain Disability Index.

b CPAQ: Chronic Pain Acceptance Questionnaire.

c PCS: Pain Catastrophizing Scale.

d PSEQ: Pain Self-Efficacy Questionnaire.

e PROMIS-29: Patient-Reported Outcomes Measurement Information System 29-item Profile.

f PROMIS-10: Patient-Reported Outcomes Measurement Information System 10-item Global Health Scale.

g GRC: Global Rating of Change Scale.

### Adverse and Positive Events

Participants recorded the adverse and positive events associated with the MBSR intervention after each session in their journals.

### Sample Size

This 2-arm external pilot trial was designed primarily to assess feasibility and inform the planning of a subsequent definitive trial, and secondarily to estimate the SD of key clinical outcomes, including the PDI. Since pilot trials are not intended for formal hypothesis testing or conventional statistical power, but rather to generate reliable estimates of feasibility and variability, we followed published recommendations [59], suggesting that a main trial powered at 90% with a 2-sided 5% significance level requires a pilot sample of approximately 25 participants per arm to detect a small (0.2) standardized effect size. We recruited 61 participants (30 MBSR and 31 control), meeting this recommended range and providing a reliable estimate of the SD for the main trial. This sample also represents 15% of our estimated main trial sample size, well above the 9% minimum recommended by the CI approach for pilot trial sample sizes [60].

### Data Analysis

Descriptive statistics were used to summarize demographic and clinical characteristics and questionnaire scores, with continuous variables reported as means and SDs and categorical variables as frequencies and percentages. Baseline characteristics were compared between groups using independent-samples *t* tests for continuous variables and chi-square tests for categorical variables, substituting the Fisher-Freeman-Halton exact test where expected cell values fell below 5.

The secondary outcomes analysis followed the ITT principle. Because 36.1% of follow-up data were missing across outcomes, linear mixed-effects models were used as the primary analytic approach, since these models use all available observations without requiring imputation of missing values [61]. For each outcome, a model was fitted with group (MBSR vs control) and assessment period (T1, T2, and T3) as fixed effects, along with their interaction, using an unstructured covariance matrix to account for the correlation between repeated assessments within participants; models were estimated by maximum likelihood, with group as the between-subject factor and assessment period as the within-subject factor. As a sensitivity analysis, these models were repeated in the subset of participants with complete data at all 3 assessment points (n=37), to assess whether findings were robust despite missing data. Whenever a statistically significant group-by-assessment period interaction was observed, post hoc comparisons using the least significant difference procedure determined between-group differences and within-group changes over time. Finally, because the GRC scale is ordinal, group differences were assessed using a Mann-Whitney *U* test, restricted to the same complete-case subset (n=37) since this measure was collected only at postintervention and could not be modeled longitudinally. All analyses of secondary outcomes were conducted on an exploratory basis to inform the design of a future definitive trial, and should not be interpreted as confirmatory evidence of intervention effects.

Additionally, a post hoc descriptive analysis was conducted to explore whether engagement with the MBSR intervention was associated with outcomes, stratifying participants into 3 tiers based on session attendance and home practice: ≥4 sessions with adequate practice (n=19), ≥4 sessions with inadequate practice (n=3), and ≤3 sessions with inadequate practice (n=8). Means and SDs were calculated at T1, T2, and T3 within each tier, without formal statistical comparisons given the small and unequal subgroup sizes. The statistical significance level was set at α=.05, and all analyses were performed using IBM SPSS (version 29; IBM Corp).

## Results

### Demographics and Clinical Variables

**Figure 1.**
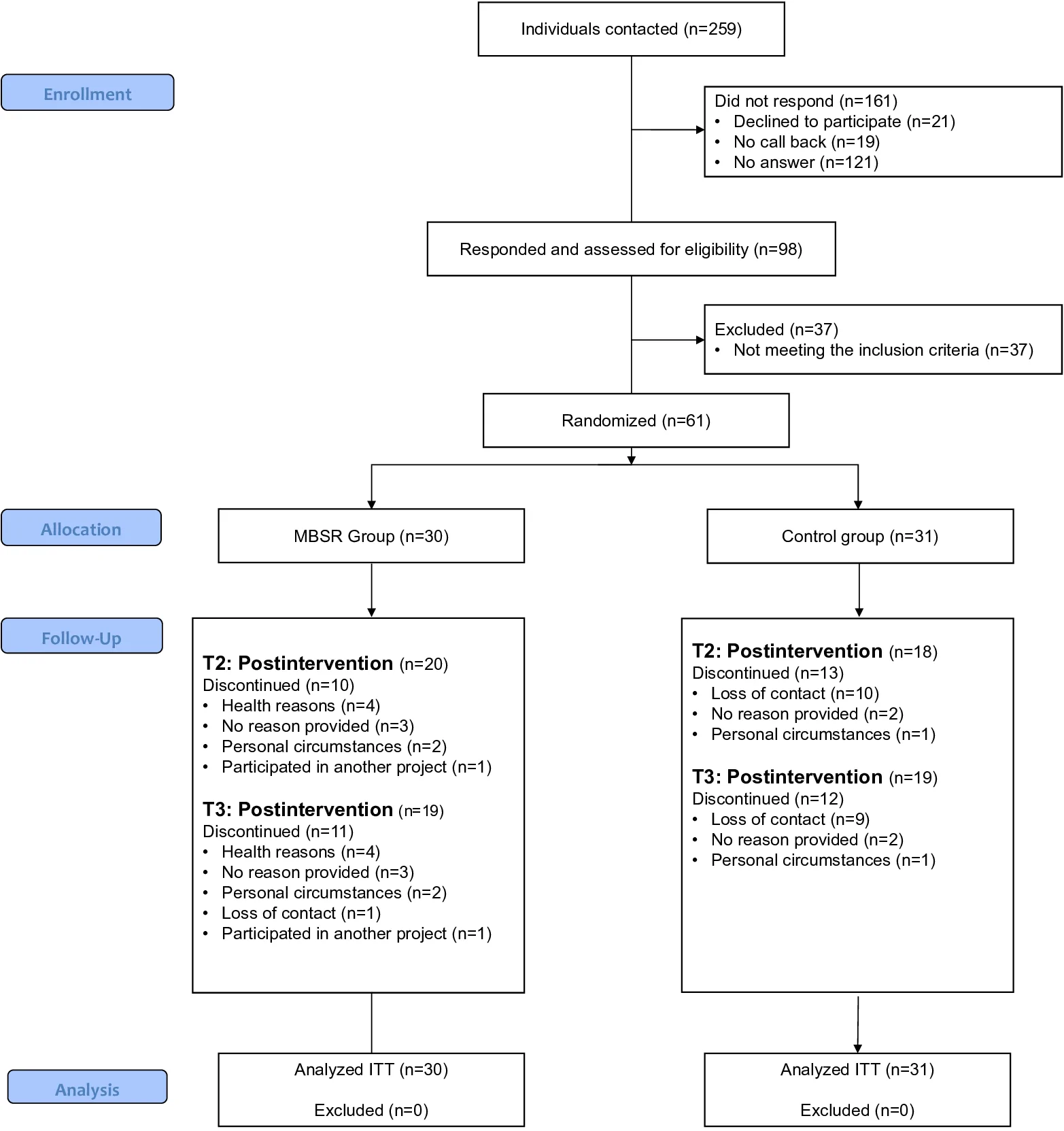
CONSORT (Consolidated Standards of Reporting Trials) flow diagram illustrating participant recruitment, eligibility screening, randomization, follow-up, and intention-to-treat (ITT) analysis in a pilot randomized controlled trial evaluating an 8-week virtual mindfulness-based stress reduction (MBSR) intervention versus a no-intervention control condition among adults with chronic pain in Quebec, Canada (May 2021-February 2022).

Of 259 individuals contacted, 161 did not respond, and 98 were assessed for eligibility; 37 were excluded for not meeting inclusion criteria (Figure 1). The remaining 61 individuals were randomized—30 to MBSR and 31 to control; their baseline demographic and clinical characteristics are presented in Table 2.

**Table 2.** Baseline demographic and clinical characteristics of participants with chronic pain enrolled in a pilot randomized controlled trial comparing an 8-week virtual mindfulness-based stress reduction (MBSR) intervention with a no-intervention control condition in Quebec, Canada (May 2021-February 2022).

Characteristics	MBSR	Control	*P* value
Demographics			
Sample size, n	30	31	—^a^
Female, n/N (**%**)	24/30 (80)	24/31 (77)	.80
Age (y), mean (SD)	56.7 (10.8)	54.4 (9.8)	.38
Marital status^b^, n/N (%)			
Single	13/27 (48.1)	15/31 (48.4)	.99
Married or common-law	14/27 (51.9)	16/31 (51.6)	.99
Employment status^b^, n/N (%)			
Employed	9/27 (33.3)	7/31 (22.6)	.13
On leave	7/27 (25.9)	15/31 (48.4)	.13
Retired	9/27 (33.3)	4/31 (12.9)	.13
Student	0/27 (0)	2/31 (6.5)	.13
Unemployed	2/27 (7.4)	3/31 (9.7)	.13
Education^b^, %			
University	14/27 (51.9)	13/31 (41.9)	.45
College or school	13/27 (48.1)	18/31 (58.1)	.45
Clinical characteristics			
Pain duration (y), mean (SD)	14.4 (11.9)	11.6 (8.2)	.27
Pain intensity in past 7 days^c^, mean (SD)	6.9 (1.5)	6.6 (1.2)	.36
Site of pain, n/N (%)			
Neck	5/30 (16.7)	1/31 (3.2)	.10
Spine	25/30 (83.3)	25/31 (80.6)	.78
Upper extremities	12/30 (40)	13/31 (41.9)	.87
Lower extremities	10/30 (33.3)	15/31 (48.4)	.23
On pain clinic waiting list, n/N (%)			
Yes	14/30 (46.7)	20/31 (64.5)	.20
No	16/30 (53.3)	11/31 (35.5)	.20
Type of pain, n/N (%)			
Musculoskeletal	26/30 (86.7)	27/31 (87.1)	.96
Neurologic	8/30 (26.7)	10/31 (32.3)	.63
Other	1/30 (3.3)	1/31 (3.2)	>.99
History of substance use, n/N (%)			
Cannabis	11/30 (36.7)	9/31 (29)	.52
Opioids	13/30 (43.3)	14/31 (45.2)	.88
Other medications	22/30 (73.3)	23/31 (74.2)	.94

a Not applicable.

b The data from the MBSR group is available for 27 participants.

c Taken from PROMIS-10 subscale 10.

In the MBSR group, 10 participants discontinued or withdrew before T2, leaving data from 20 participants at that time point. Reasons for discontinuation included employment-related problems (n=2), involvement in another project, significant pain, health-related issues, exhaustion, and inability to keep up (1 participant each), with 3 discontinuing without providing a reason. At T3, data were collected from 19 MBSR participants, as 1 additional participant, along with all those who had already discontinued at T2, did not respond to the questionnaires.

In the control group, 18 participants responded to outcome measures at T2. Of those who discontinued, 10 did not complete the questionnaires, 1 cited a personal reason, and 2 gave no specific reason. Moreover, 1 participant who had not responded at T2 resumed contact with the researcher by email at T3, bringing the control group’s T3 total to 19 participants.

### MBSR Intervention Fidelity

Average intervention duration was 1860 (SD 1377.2) minutes. A total of 19 participants attended at least 4 sessions and completed at least half the required individual practice time, 3 attended at least 4 sessions but completed less than half the required practice, and 8 attended 3 or fewer sessions with less than half the required practice completed.

### Primary Outcomes: Feasibility Parameters

Table 3 presents the feasibility parameters. None of the 3 prespecified thresholds were met: recruitment (61/259, 23.5% vs target 30%), retention (37/61, 60.7% vs target 80%), and adherence (19/30, 63.3% vs target 80%). Among the 61 randomized participants, average questionnaire administration time was 36 (SD 12.2) minutes for MBSR and 36 (SD 10.6) minutes for control. Participants were also required to maintain a daily practice journal throughout the intervention. Of the 240 expected journal entries, 164 (68.3%) were completed by the 30 participants.

**Table 3.** Feasibility outcomes of the pilot randomized controlled trial evaluating an 8-week virtual mindfulness-based stress reduction (MBSR^a^) intervention versus a no-intervention control condition among adults with chronic pain in Quebec, Canada (May 2021-February 2022), including recruitment, intervention adherence, retention, and questionnaire completion rates.

Parameter and aspect	Rate, %
Recruitment rate	
Individuals who agreed to participate out of individuals who were contacted	23.5
Adherence to intervention	
≥4 sessions and half or more required individual practice time	63.3
≥4 sessions and less than half required individual practice time	10
≤3 sessions and less than half required individual practice time	26.6
Retention rate of study participants	
Total participants who attended all 3 months of the study	60.7
Total participants who attended all 3 months of the study in the MBSR^a^ group	63.3
Total participants who attended all 3 months of the study in the control group	58.1
T1 participation (total)	100
T2 participation (total)	62.3
T3 participation (total)	62.3
T1 participation (MBSR group)	100
T1 participation (control group)	100
T2 participation (MBSR group)	66.7
T2 participation (control group)	58.1
T3 participation (MBSR group)	63.3
T3 participation (control group)	61.3

a MBSR: mindfulness-based stress reduction.

### Secondary Outcomes

#### Overview

The following secondary outcomes are reported as exploratory analyses (Table 4) to inform the design of a future definitive trial. Given that this pilot trial was not powered to detect intervention effects, these findings should be interpreted cautiously and not considered confirmatory evidence of treatment effect.

**Table 4.** Descriptive statistics (mean and SE) and linear mixed model results for clinical outcome measures among all participants with available data at each assessment (n=61; MBSR=30, control=31), assessed at baseline (T1), postintervention (T2), and 3-month follow-up (T3), among adults with chronic pain randomized to an 8-week virtual mindfulness-based stress reduction (MBSR) intervention or a no-intervention control condition in a pilot randomized controlled trial conducted in Quebec, Canada (May 2021 to February 2022).

Outcome	MCID^a^ (study citation)	Experimental (n=30). mean (SE)			Control (n=31), mean (SE)			Linear mixed model		
		T1	T2	T3	T1	T2	T3	Time	Group	Time×group
Pain Disability Index (overall)	8.5 to 9.5 [62]	36 (1.7)	31.8 (2.6)	24.0 (2.7)^b,c,d,e^	39.1 (1.7)	38.4 (2.7)	36.0 (2.7)^d^	<.001	.01	.03
Pain Catastrophizing Scale (overall)	8 to 11 [63]	26.6 (1.7)	18.5 (2.1)	17.4 (2.1)^e^	29.3 (1.7)	25.6 (2.1)	23.0 (2.1)	<.001	.05	.11
Chronic Pain Acceptance Questionnaire (overall)	—^f^	29 (1.0)	27.8 (1.2)	28.7 (1.2)	28.9 (1.0)	28.8 (1.2)	30.4 (1.2)	.22	.53	.57
Pain Self-Efficacy Questionnaire (overall)	5.5 to 8.5 [64]	35.8 (1.7)	40.8 (1.7)	39.7 (2.2)	32.1 (1.7)	33.1 (1.7)	35.5 (2.2)	.07	.02	.20
PROMIS-10^g^ Global Health	2 to 6 [65]									
T-Score Physical health		35.4 (0.8)	40.1 (1.0)^e,h,i^	37.7 (1.1)^b,e^	34.6 (0.8)	34.7 (1.0)^i^	35.2 (1.1)	.003	.01	.003
T-Score Mental health		41.2 (1.3)	45.2 (1.2)^e^	44.9 (1.3)^e^	39.6 (1.2)	40.4 (1.3)^e^	40.6 (1.3)	.01	.03	.16
Global health		2.6 (0.1)	2.8 (0.1)^i^	2.8 (0.1)	2.3 (0.1)	2.2 (0.1)^i^	2.5 (0.1)^c^	.20	.04	.02
Social activities and role		2.7 (0.1)	2.8 (0.2)	2.9 (0.1)	2.1 (0.1)	2.5 (0.2)	2.4 (0.1)	.06	.04	.73
Pain intensity (0‐10)		6.9 (0.2)	5.7 (0.3)	5.7 (0.3)	6.5 (0.2)	6.4 (0.3)	5.9 (0.3)	.004	.60	.13
PROMIS-29^j^ T-Score	2 to 6 [65]									
Pain interference		64.4 (0.9)	59.9 (0.9)^e^	60.2 (1.3)^e^	66.3 (0.9)	63.8 (1.0)	64.4 (1.3)	<.001	.01	.25
Participation in social activities		40.2 (1.2)	46.4 (1.5)^e,h,i^	41.7 (1.6)^b^	37.5 (1.2)	39.8 (1.5)^e,i^	41.3 (1.6)^c,e^	.001	.06	.02
Physical function		38.9 (1.0)	39.6 (0.9)	39.9 (1.3)	36.2 (1.0)	37.1 (0.9)	36.6 (1.3)	.14	.06	.84
Fatigue		61.9 (1.2)	57.6 (1.7)^e^	56.7 (1.8)^e^	62.9 (1.2)	61.7 (1.8)	60.5 (1.8)	.01	.10	.38
Anxiety or fear		61.7 (1.3)	54.8 (1.6)^e^	55.6 (1.7)^e^	62.6 (1.3)	60.5 (1.7)	58.5 (1.7)^e^	<.001	.10	.10
Sleep disturbance		57.8 (1.5)	53.3 (1.8)^e^	53.1 (2.0)^e^	61.4 (1.4)	58.7 (1.9)	57.1 (2.1)^e^	.02	.04	.73
Depression or sadness		59.1 (1.3)	54.8 (1.7)^e^	53.8 (1.7)^e^	59.8 (1.3)	60.1 (1.8)	57.8 (1.7)	.004	.09	.18

a MCID: minimally clinically important difference.

b Post hoc within-group differences with least significant difference corrections within T2 − T3.

c Post hoc within-group differences with least significant difference corrections within T1 − T3.

d Post hoc between-group differences with least significant difference corrections at T3.

e Minimum clinically important difference from baseline values.

f Not available.

g PROMIS-10: Patient-Reported Outcomes Measurement Information System-10.

h Post hoc within-group differences with least significant difference corrections within T1 − T2.

i Post hoc between-group differences with least significant difference corrections at T2.

j PROMIS-29: Patient-Reported Outcomes Measurement Information System-29.

#### PDI

A linear mixed-effects model showed significant main effects of group (*F*_1, 58.1_=6.72, *P*=.01) and assessment period (*F*_2, 38.8_=11.24, *P*<.001) as well as a group-by-assessment period interaction (*F*_2, 38.8_=3.90, *P*=.03). Post hoc analyses showed lower PDI scores in the MBSR group than in the control group at T3 (*P*=.003). Within the MBSR group, PDI scores decreased significantly from T1 to T3 (*P*<.001) and from T2 to T3 (*P*=.004), whereas no significant changes were observed in the control group.

#### PCS

There was a significant main effect of assessment period (*F*_2, 39.1_=22.70, *P*<.001) but no significant main effect of group (*F*_1, 59.7_=4.01, *P*=.05) or group-by-assessment period interaction (*F*_2, 39.1_=2.32, *P*=.11).

#### CPAQ

There were no significant effects of assessment period (*F*_2, 39.1_=1.55, *P*=.22) or group (*F*_1, 54.6_=0.39, *P*=.53). Additionally, there was no significant interaction between group-by-assessment period (*F*_2, 39.1_=0.56, *P*=.57.

#### PSEQ

There was a significant main effect of group (*F*_1, 55.5_=5.87, *P*=.02), but no significant main effect of assessment period (*F*_2, 44.1_=2.84, *P*=.07) or group-by-assessment period interaction (*F*_2, 44.1_=1.68, *P*=.20).

#### PROMIS-10

##### Physical Health T-Score

There were significant main effects of group (*F*_1, 48.7_=6.43, *P*=.01) and assessment period (*F*_2, 39.7_=6.82, *P*=.003), as well as a significant group-by-assessment period interaction (*F*_2, 39.7_=6.57, *P*=.003). Post hoc analyses showed that the MBSR group had significantly higher physical health T-scores than the control group at T2 (*P*<.001). Within the MBSR group, scores improved significantly from T1 to T2 (*P*<.001) and from T2 to T3 (*P*=.04), whereas no significant changes were observed in the control group.

##### Mental Health T-Score

There were significant main effects of group (*F*_1, 61.9_=4.96, *P*=.03) and assessment period (*F*_2, 43.0_=4.90, *P*=.01). The group-by-assessment period interaction was not significant (*F*_2, 43.0_=1.94, *P*=.16.

##### Social Activities and Role

There was a significant main effect of group (*F*_1, 51.6_=4.34, *P*=.04). The main effect of assessment period (*F*_2, 40.6_=3.07, *P*=.06) and the group-by-assessment period interaction (*F*_2, 40.6_=0.31, *P*=.73) were not significant.

##### Global Health

There were significant main effects of group (*F*_1, 52.4_=4.54, *P*=.04) and a significant group-by-assessment period interaction (*F*_2, 40.2_=4.56, *P*=.02). The main effect of assessment period was not significant (*F*_2, 40.2_=1.70, *P*=.20). Post hoc analyses showed higher global health scores in the MBSR group than the control group at T2 (*P*=.006). Within-group comparisons showed a significant reduction in scores from T1 to T3 in the control group (*P*=.01), whereas no significant changes were observed in the MBSR group.

##### Pain Intensity

There was no significant main effect of group (*F*_1, 48.4_=0.29, *P*=.60). The main effect of assessment period was significant (*F*_2, 41.2_=6.33, *P*=.004), but the group-by-assessment period interaction was not significant (*F*_2, 41.2_=2.16, *P*=.13).

### PROMIS-29

#### Pain Interference T-Score

There were significant main effects of group (*F*_1, 54.2_=7.08, *P*=.01) and assessment period (*F*_2, 40.0_=16.13, *P*<.001). The group-by-assessment period interaction was not significant (*F*_2, 40.0_=1.46, *P*=.25.

#### Anxiety or Fear T-Score

There was no significant main effect of group (*F*_1, 60.1_=2.72, *P*=.10). The main effect of assessment period was significant (*F*_2, 40.5_=13.10, *P*<.001), but the group-by-assessment period interaction was not significant (*F*_2, 40.5_=2.50, *P*=.10).

#### Depression or Sadness T-Score

There was no significant main effect of group (*F*_1, 52.8_=2.93, *P*=.09). The main effect of assessment period was significant (*F*_2, 40.2_=6.32, *P*=.004), but the group-by-assessment period interaction was not significant (*F*_2, 40.2_=1.82, *P*=.18).

#### Fatigue T-Score

There was no significant main effect of group (*F*_1, 54.3_=2.83, *P*=.10). The main effect of assessment period was significant (*F*_2, 41.2_=4.74, *P*=.01), but the group-by-assessment period interaction was not significant (*F*_2, 41.2_=1.00, *P*=.38)*.*

#### Sleep Disturbance T-Score

There were significant main effects of group (*F*_1, 44.3_=4.35, *P*=.04) and assessment period (*F*_2, 40.0_=4.42, *P*=.02). The group-by-assessment period interaction was not significant (*F*_2, 40.0_=0.32, *P*=.73.

#### Physical Function

There were no significant main effects of group (*F*_1, 58.6_=3.78, *P*=.06) or assessment period (*F*_2, 40.2_=2.06, *P*=.14). The group-by-assessment period interaction was also not significant (*F*_2, 40.2_=0.17, *P*=.84.

#### Participate in Social Activities T-Score

There was no significant main effect of group (*F*_1, 55.5_=3.56, *P*=.06) but significant main effects of assessment period (*F*_2, 40.9_=8.22, *P*=.001) and a significant group-by-assessment period interaction (*F*_2, 40.9_=4.43, *P*=.02). Post hoc analyses showed higher scores in the MBSR group than the control group at T2 (*P*=.005). Within-group comparisons showed significant changes in the MBSR group from T1 to T2 (*P*<.001) and T2 to T3 (*P*=.002), and in the control group from T1 to T3 (*P*=.004).

### GRC

At postintervention, the MBSR group reported significantly greater perceived improvement on the GRC than the control group (MBSR: mean 3.05, SD 1.43; n=19; and control: mean 0.50, SD 2.09; n=18), as indicated by a Mann-Whitney *U* test (*U*=50.5, *Z*=−3.79, *P*<.001).

### Sensitivity Analysis

Results from the complete-case sensitivity analysis, restricted to participants with data at all 3 assessment points (n=37), are presented in Multimedia Appendix 2. Findings were generally consistent between the 2 analyses. The significant group-by-assessment period interactions observed for the PDI, PROMIS-10 physical health, PROMIS-10 global health, and participation in social activities were replicated in the complete-case sample. Several overall group main effects that were significant in the primary analysis, including PROMIS-10 mental health, global health, social activities and role, and sleep disturbance, did not reach significance in the smaller complete-case sample, likely reflecting reduced statistical power rather than meaningful difference in effect. Conversely, the group effect for physical function and the interaction for participation in social activities reached significance only in the complete-case analysis. Overall, the pattern of findings did not meaningfully diverge between the 2 analyses, supporting the robustness of the primary results despite missing data.

### Exploratory Analysis of Intervention Engagement and Outcomes

A post hoc descriptive analysis explored whether engagement with the MBSR intervention was associated with outcomes across the 3 engagement tiers (Multimedia Appendix 3). Among participants who attended 4 or more sessions and completed adequate home practice (n=19), consistent improvements were observed across most outcomes from T1 to T3, several of which met or approached MCID thresholds. Among participants who attended 4 or more sessions but completed inadequate home practice (n=3), a similar directional trend was observed for some outcomes, although the very small subgroup size precludes any meaningful interpretation. Among participants who attended fewer than 4 sessions (n=8), follow-up data were available for only 1 participant at T2 and T3, rendering group-level estimates uninterpretable. Given these limitations, no formal statistical comparisons were conducted between engagement tiers, and findings are reported for descriptive purposes only.

### Adverse and Positive Events

During the intervention, MBSR group participants reported a total of 26 adverse events through self-reported journals. The most common adverse events were fatigue during exercises (7/26, 26.9%), pain during exercises (6/26, 23.1%), and stress, agitation, and impatience during yoga (4/26, 15.4%). Other adverse events included feelings of judgment and frustration (3/26, 11.5%), dizziness and shortness of breath during yoga (2/26, 7.7%), headache or migraine (2/26, 7.7%), thirst during yoga (1/26, 3.8%), and flashbacks during mindfulness (1/26, 3.8%). Most events occurred during the first session. Participants could also contact the therapist by email to report adverse events or concerns. At the start of each session and during group discussions, participants were asked about any difficulties; however, no adverse events were reported through these channels.

In total, 18 participants in the MBSR group reported a total of 41 positive events, the most common being pain reduction or control (12/41, 29.3%), a sense of well-being (7/41, 17.1%), increased energy (6/41, 14.6%), decreased negative thoughts (6/41, 14.6%), a sense of control and rediscovery of life (5/41, 12.2%), and decreased anxiety (5/41, 12.2%).

## Discussion

### Principal Findings

The primary finding of this pilot RCT was that recruitment, retention, and adherence did not meet the prespecified feasibility thresholds, indicating that the study design requires modification before a definitive trial. Exploratory analyses of clinical outcomes were conducted to inform the design of a future trial and should be interpreted cautiously, as this pilot study was not designed or powered to evaluate intervention effectiveness. Although trends toward improvements in pain disability, physical health, global health, participation in social activities, and GRC were observed among participants receiving MBSR, these findings require confirmation in a future adequately powered trial.

### Feasibility of MBSR

Assessing feasibility is a core purpose of pilot studies, helping identify the practicality of delivering an intervention and anticipate challenges [66]. In this study, the feasibility parameters were not met, indicating the need for design modifications. We observed a lower-than-expected recruitment rate of 23.5% among individuals with CP who were initially contacted. In our view, a key factor influencing recruitment in this study is the complexity of the consent process. The detailed information provided about the intervention, and particularly the potential burden it could place on individuals with CP, may have discouraged participation [67]. Some individuals may also have been reluctant to enroll due to the possibility of being assigned to a control group, receiving no intervention while still being expected to complete follow-up assessments. To improve recruitment, we suggest simplifying the consent form to enhance understanding of the procedure [68]. In addition, the virtual format itself may have influenced recruitment, as some individuals with CP may feel less comfortable with videoconference-based group sessions or may lack confidence with technology, which has been reported as a barrier to participation in virtual health interventions [69]. These technology-related factors may therefore have contributed to the modest recruitment rates observed. Additionally, although we recruited both individuals on a waiting list and those who were not on a waiting list and were not receiving pain services, recruitment from pain clinic waiting lists may have introduced a potential mismatch between participants’ expectations and the nature of a time-intensive, skill-based intervention such as MBSR. Individuals seeking more immediate symptom relief may have found the active practice requirements of MBSR more challenging, which could have influenced engagement and retention. The proportion of participants recruited from a waiting list did not differ significantly between groups, suggesting recruitment source alone is unlikely to explain the differential attrition observed between arms. However, our pilot sample was not powered to formally examine whether attrition differed according to recruitment source. Future trials may benefit from recruiting individuals who have previously attended pain rehabilitation programs and continue to experience long-term difficulties, as prior experience with pain self-management may facilitate engagement with mindfulness-based interventions.

Our pilot study also had lower-than-expected retention rates (60.7% vs 80%). Notably, participants in the control group had higher dropout rates at T2 (41.9% vs 33.3%) and T3 (38.7% vs 36.7%) compared with participants in the MBSR group; however, these differences were not statistically evaluated. Several factors may explain the lower-than-expected retention. First, retention in the intervention group may have been influenced by factors unique to virtual delivery, such as reduced sense of group cohesion, home-based distractions, and “Zoom fatigue,” which may partially explain the retention challenges observed in our study [70]. Employment-related constraints and personal demands may also have influenced sustained engagement, as some MBSR participants who discontinued reported challenges related to work demands, exhaustion, or difficulty keeping up with the intervention.

Fatigue may have been an important consideration for engagement, consistent with its being the most commonly reported adverse event. Although breaks were provided within sessions to help mitigate fatigue, additional strategies may be needed to better accommodate participants experiencing fatigue. Second, the absence of intervention in the control group may have increased attrition, as participants may have had unmet expectations and felt less motivated without support, feedback, or interactions with the research team. Third, participants in the control group may have found it challenging to adhere to the study protocol if they did not perceive direct benefits, leading them to seek alternative treatments outside the study [71]. To address this, we propose a waitlist-control design, in which control participants receive the intervention after the initial study period, which may improve retention by increasing motivation to remain in the study [72]. We also plan to offer financial incentives to all participants [73] and increase personalized communication, particularly with the control group, so that all participants feel engaged and valued throughout the study [74]. The administration of numerous questionnaires during follow-up assessments may have increased participant burden, potentially contributing to fatigue, frustration, and dropout [75]. As a modification for the larger trial, we plan to reduce the number of questionnaires used, since the pilot trial used both PROMIS-29 and PROMIS-10, and PROMIS-29 provides a more comprehensive assessment of HRQoL, encompassing the aspects that PROMIS-10 covers only briefly. Excluding PROMIS-10 from the future design would therefore reduce both questionnaire redundancy and overall respondent burden.

Regarding adherence to the MBSR intervention, of the 30 participants in the MBSR group, 19 (63.3%) attended 4 or more sessions and completed at least half of the required individual practice time, lower than our target of 80%. Two factors might have contributed to this reduced adherence. First, part of the trial coincided with the lifting of COVID-19 quarantine measures, easing travel restrictions, and the arrival of spring in 2022. Many participants expressed a desire to go outdoors rather than stay indoors and complete online sessions. Second, some participants experienced logistical issues during the MBSR intervention that may have affected their adherence. In particular, several individuals lost access to their daily journal, a key component of the intervention, which may have disrupted their engagement with home practice tracking and contributed to overall disengagement. To improve adherence in future studies, we suggest implementing the following modifications. First, offering greater flexibility in scheduling virtual MBSR sessions by allowing participants to choose from time slots could better accommodate individual preferences and increase participation [76,77]. Second, streamlining the journaling process or transitioning to a more user-friendly digital platform may reduce participant burden and enhance adherence [78]. Beyond these logistical considerations, group size may also have influenced participant engagement. Although the MBSR intervention was delivered by an experienced therapist with formal training in MBSR, and participants were divided into 2 parallel groups of 15, a size the therapist found manageable, the virtual group format may still have presented challenges related to group cohesion, individual visibility, and participant experience, particularly for individuals managing persistent pain, fatigue, and emotional distress. Future trials should explore optimal group sizes to further support group cohesion and participant well-being.

It is also worth considering whether the standard 8-week MBSR protocol requires adaptation for individuals with CP. The MBSR program used here followed the core format and content described by Kabat-Zinn [39]. Originally developed for a broad range of stress-related conditions, this protocol may place demands on participants, including extended session duration, intensive home practice requirements, and sustained attentional focus, that are particularly challenging for individuals with CP. Future studies could explore adaptations such as shorter or more flexible session formats, a more gradual introduction to home practice, and pain-specific psychoeducational content to better meet the needs of this population, potentially improving adherence and retention. Future iterations may also benefit from enhancing between-session resources, such as recorded practice videos and self-report journals, to better support home practice and engagement.

Relatedly, a post hoc descriptive analysis further explored patterns in outcomes across 3 intervention engagement tiers. Participants who attended 4 or more sessions and completed adequate home practice demonstrated the most consistent descriptive trends toward improvement across pain-related and well-being outcomes, with some changes reaching or approaching previously reported MCID thresholds, suggesting that full engagement with both the supervised and home practice components may be important for achieving meaningful clinical benefit. This is consistent with existing literature indicating that greater home practice is associated with improved psychological and symptom-related outcomes in MBSR [79], and that there is a small but significant association between the extent of formal practice and positive intervention outcomes across diverse populations [80]. However, given the very small number of participants in the lower-engagement tiers and the near-complete absence of follow-up data in the lowest-engagement group, these findings are hypothesis-generating only and should not be interpreted as evidence of a dose-response relationship. Future trials should prospectively capture attendance and home practice data and be adequately powered to formally examine whether engagement influences intervention outcomes.

### Pain Disability

In individuals with CP, pain-related disability significantly impacts physical functioning, emotional well-being [81], and the ability to perform daily activities [82]. This disruption can negatively affect personal, social, and occupational life, ultimately reducing overall quality of life [83]. Recognizing and addressing pain-related disability is therefore essential in the comprehensive management of CP. Preliminary exploratory analyses suggested that participants who received MBSR showed a significant reduction in pain disability scores by 3-month follow-up compared with the control group, although these findings should be interpreted cautiously given the underpowered sample. Within the MBSR group, reductions in pain disability emerged progressively, with significant decreases from baseline to 3-month follow-up and from postintervention to 3-month follow-up, whereas no significant within-group changes were observed in the control group.

The MBSR group also showed a mean reduction in pain disability at 3-month follow-up (mean difference [MD] T3 − T1: −12), exceeding the 8.5 to 9.5-point threshold typically considered clinically meaningful for individuals with CP [62], compared with a smaller reduction in the control group (MD T3 − T1: −3.1). Previous research has linked reductions in pain disability to improved quality of life and enhanced cognitive and behavioral coping strategies [57], underscoring the potential relevance of MBSR for pain management.

### Pain Catastrophizing

In addition to contributing to disability, CP is also strongly associated with pain catastrophizing, a pattern of thinking characterized by magnifying the threat of pain, dwelling on it, and feeling helpless in response. Preliminary exploratory analyses revealed no significant group difference or interaction in PCS scores, although the MBSR group showed a numerically greater reduction (MD T3 − T1: −9.2) compared with the control group (MD T3 − T1: −6.3). This reduction fell within the 8 to 11-point threshold typically considered clinically meaningful for individuals with CP [63], although this finding is hypothesis-generating only, given the nonsignificant group and interaction effects, and should be interpreted with caution.

### Pain Self-Efficacy

Beyond its association with pain catastrophizing, CP is also linked to reduced pain self-efficacy, the belief that individuals have in their ability to function effectively despite experiencing pain [64]. Enhancing pain self-efficacy has been proposed as a psychological resource that promotes physical function, reduces fear avoidance, and ultimately leads to improved pain management outcomes [54,84]. Studies have shown that changes in self-efficacy levels can reflect patterns of success and failure, with higher self-efficacy encouraging individuals to take on new challenges and low self-efficacy hindering pursuit of new opportunities [85,86]. In this study, no significant group-by-time interaction was observed for PSEQ scores. At postintervention, the MBSR group showed a numerically greater improvement in PSEQ (MD T2 − T1: 5.0 points), compared with the control group (MD T2-T1: 1 point), though by 3-month follow-up this difference had narrowed considerably (MD T3 − T1: +3.82 for MBSR vs+3.4 for control), and neither group’s change reached the threshold typically considered clinically meaningful.

This preliminary improvement in pain-related self-efficacy was also reflected in the GRC questionnaire, which assessed participants’ perceptions of their ability to manage pain following the intervention. The significant improvement in the GRC among those who received MBSR compared with the control group suggests that this intervention may have facilitated the development and implementation of adaptive coping strategies, reducing the impact of pain on their daily activities. Moreover, enhancing self-efficacy levels could positively affect the psychological well-being of individuals with CP. Notably, positive experiences reported by individuals in the MBSR group included a pain reduction or control (12/41, 29.3%), and a sense of well-being (7/41, 17.1%).

### HRQoL

The MBSR intervention was also associated with improvements across some domains of HRQoL, particularly PROMIS-10 physical health and global health, and PROMIS-29 participation in social activities, each of which showed a significant group-by-time interaction favoring MBSR. The improvement in physical health may be attributable, in part, to the observed reductions in pain-related disability, potentially enabling individuals to engage more readily in daily activities despite experiencing CP. Similarly, the improvement in participation in social activities is noteworthy, as CP often predisposes individuals to social isolation and difficulties in maintaining interpersonal relationships [87], and this finding may suggest that MBSR supported greater social engagement [88]. These findings should nonetheless be interpreted cautiously, given the pilot design and limited sample size.

With regard to other subdomains assessed by the PROMIS questionnaires, although no significant interactions were observed, PROMIS-29 pain interference showed a significant overall difference between groups, without a significant change in this difference over time, and this effect remained consistent in the complete-case sensitivity analysis. PROMIS-10 mental health, social activities and role, and PROMIS-29 sleep disturbance also showed significant overall between-group differences in the primary analysis, although these did not reach significance in the complete-case sensitivity analysis and should therefore be interpreted with particular caution. PROMIS-10 pain intensity, and PROMIS-29 fatigue, anxiety or fear, and depression or sadness improved significantly over time, but with no significant difference between groups, indicating these improvements were not specific to MBSR.

These outcomes remain clinically relevant, as individuals with CP commonly experience depression, anxiety, fatigue, and sleep disturbance, all of which are closely linked to greater pain severity and disability [89]. Mindfulness has been suggested to reduce the subjective experience of pain, which may in turn lessen its psychological consequences and support coping [90]. Sleep disturbance also has a bidirectional relationship with CP, whereby pain disrupts sleep while poor sleep further amplifies pain perception and psychological distress [91]. By potentially decreasing negative emotional states and supporting coping strategies, MBSR may contribute to broader improvements in well-being and HRQoL, although this remains to be established. Nonetheless, given the exploratory nature of these analyses, these are merely hypothesis generating and the trial’s limited power, these associations require confirmation in an adequately powered trial.

Overall, the primary goal of this feasibility pilot RCT was to evaluate the viability of the study design, methods, recruitment strategies, and MBSR intervention for a larger trial, rather than to detect significant effects, given that the sample size was not calculated for this purpose. This study also allowed us to identify key challenges and refine the protocol to ensure the success of a full-scale trial. Nevertheless, preliminary exploratory analyses on secondary outcomes revealed improvements in the PDI, the PROMIS-29 participation in social activity subset, the PROMIS-10 physical health and global health subsets, and the GRC in the MBSR group compared with the control group. Some outcomes, such as PCS, did not reach statistical significance despite showing a numerical change that met minimum thresholds for clinically important difference reported in the literature. Nonetheless, these findings should be interpreted cautiously given that the study was not powered to detect changes in these outcomes. A complete-case sensitivity analysis, restricted to participants with data at all 3 assessment points, was also conducted to assess the robustness of these findings to the handling of missing data, and results concerning the interaction between group and assessment period were consistent with the primary analysis for these 4 outcomes. Furthermore, the engagement-stratified descriptive analysis suggested that participants who fully engaged with both the supervised sessions and home practice components showed the most consistent improvements across outcomes, underscoring the importance of addressing adherence barriers in the planned definitive trial.

### Limitations

Despite the novelty of this study, several limitations should be acknowledged. First, as a pilot trial with a small sample size, the study was not designed or powered to detect intervention effects, and the findings should therefore be interpreted as preliminary. The limited number of participants may also affect the representativeness of the sample and the generalizability of the findings. An additional consideration is that 2 optional follow-up sessions were offered after the intervention, in response to participants’ request for continued support. As these sessions were not prespecified and attendance was not systematically recorded across both cohorts, their influence on longer-term outcomes could not be precisely quantified. Although no new content was delivered, the sessions may have reinforced adherence and contributed to maintenance of effects at the 3-month follow-up. Participants also independently formed an unmoderated peer-support group during this period, adding a further uncontrolled source of ongoing engagement outside the study protocol. These factors warrant caution when interpreting the 3-month findings. Second, the exploratory analyses included multiple secondary outcomes without adjustment for multiple comparisons, increasing the risk of false-positive findings. These results should therefore be interpreted cautiously. Third, participant attrition during the follow-up period may have introduced bias and affected the integrity of the ITT analysis. However, a complete-case sensitivity analysis restricted to participants with data at all 3 assessment points showed broadly consistent findings, suggesting the primary results were not substantially driven by attrition. It is also important to note that the study was conducted during the period following the lifting of COVID-19 restrictions, which may have contributed to participant dropout. Additionally, employment status was not formally examined as a potential moderator of session attendance or home practice adherence. Although some participants cited employment-related difficulties as a reason for discontinuation, the extent to which work-related demands influenced intervention engagement could not be determined. Future trials should prospectively examine whether employment status and associated time constraints influence adherence and retention among employed participants. Furthermore, adverse event reporting relied on self-reported journals without a structured reporting format and did not systematically capture the severity, relatedness to the intervention, duration, management, or whether individual events contributed to withdrawal. Future trials should incorporate structured adverse event reporting tools to capture these elements systematically and enable more comprehensive safety evaluations. Finally, assessor blinding was not formally evaluated, which should be considered when interpreting the findings.

To generate more robust and conclusive evidence on the effects of the MBSR intervention, we plan to replicate the study with a larger sample and an improved design that addresses feasibility challenges. Doing so will enhance the validity and reliability of the findings and support more confident inferences about the effectiveness of MBSR in this population.

### Conclusion

This pilot trial identified important feasibility challenges, with recruitment (23.5%), retention (60.7%), and adherence (63.3%) falling below pre-specified thresholds. These findings indicate that modifications to study design and delivery procedures, including simplification of the consent process, targeted recruitment strategies, and consideration of a waitlist-control design, are needed before proceeding to a definitive trial. Exploratory analyses suggested improvements in pain disability and HRQoL subdomains, including physical health and social activity participation, among participants receiving MBSR compared with the no-intervention control condition. However, these findings should be interpreted cautiously, as the pilot trial was not designed or powered to evaluate intervention effectiveness. Exploratory findings related to GRC in pain self-efficacy are consistent with the potential role of mindfulness-based interventions in supporting pain self-management and coping, but require confirmation in future adequately designed trials. These results provide valuable insights into the delivery of a virtual MBSR intervention for individuals with CP in a Quebec setting and inform the refinement of a future RCT.

## Supplementary material

10.2196/98632 Multimedia Appendix 1 Detailed steps performed during the virtual mindfulness-based stress reduction intervention.

10.2196/98632 Multimedia Appendix 2 Descriptive statistics and linear mixed model results as a sensitivity analysis primary analysis of clinical outcome measures among participants with complete data at all 3 assessment points.

10.2196/98632 Multimedia Appendix 3 Post hoc descriptive analysis of outcome measures by intervention engagement tier at baseline (T1), postintervention at 8 weeks (T2), and 3-month follow-up (T3) among MBSR participants.

10.2196/98632 Checklist 1 CONSORT checklist.

## Abbreviations

CONSORT: Consolidated Standards of Reporting Trials
CP: chronic pain
CPAQ: Chronic Pain Acceptance Questionnaire
GRC: Global Rating of Change Scale
HRQoL: health-related quality of life
ITT: intention-to-treat
MBSR: mindfulness-based stress reduction
MD: mean difference
PCS: Pain Catastrophizing Scale
PDI: Pain Disability Index
PROMIS-10: Patient-Reported Outcomes Measurement Information System-10
PROMIS-29: Patient-Reported Outcomes Measurement Information System-29
PSEQ: Pain Self-Efficacy Questionnaire
RCT: randomized controlled trial
