# Supplementary material for: Feasibility of a Virtual Mindfulness-Based Stress Reduction Intervention for Individuals With Chronic Pain: Pilot Randomized Controlled Trial
Source: JMIR Form Res. 2026 Sep 23;10:e98632. doi: 10.2196/98632 (PMC13600662; doi:10.2196/98632)
Supplement: Supplementary file 1 [file formative-v10-e98632-s001.pdf]

Table. Detailed steps performed during the virtual Mindfulness based stress reduction intervention

Session	Component
Session 1	10 min: Guided meditation<br>30 min: Definition of mindfulness<br>10 min: Exercise 1: Mindful eating<br>10 min: Exercise 2. Breathing mindfully<br>10 min: Break<br>20 min: Definition of the body scan technique and exercise<br>30 min: Group sharing of effects, questioning and deepening of the material taught (group discussion for qualitative collection)<br>10 min: Guided meditation
Session 2	10 min: Guided meditation<br>30 min: Explanation of the role of perception in well-being<br>10 min: Exercise 1. Recognizing your thoughts<br>10 min: Exercise 2. Body scan<br>10 min: Break<br>20 min: Explanation of the role of perception in well-being (continued)<br>30 min: Group sharing of effects, questioning and deepening of the material taught (group discussion for qualitative collection)<br>10 min: Guided meditation
Session 3	10 min: Guided meditation<br>30 min: The importance of being in the present moment<br>10 min: Exercise 1: Sitting meditation<br>10 min: Exercise 2. Moving meditation<br>10 min: Break<br>20 min: Introduction to hatha yoga technique and exercise<br>30 min: Group sharing of effects, questioning and deepening of the material taught (group discussion for qualitative collection)<br>10 min: Guided meditation
Session 4	10 min: Guided meditation<br>30 min: The role of concentration<br>10 min: Exercise 1: Sitting meditation<br>10 min: Exercise 2. Hatha yoga<br>10 min: Break<br>20 min: The role of concentration (continued)<br>30 min: Group sharing of effects, questioning and deepening of the material taught (group discussion for qualitative collection)<br>10 min: Guided meditation
Session 5	10 min: Guided meditation<br>30 min: Pain, emotions and their impact<br>10 min: Exercise 1. Recognizing our signs of pain<br>10 min: Exercise 2. Recognizing our emotions and their impact<br>10 min: Break<br>20 min: Exercise 3. Body sweeping<br>30 min: Group sharing of effects, questioning and deepening of the material taught (group discussion for qualitative collection)<br>10 min: Guided meditation

Session 6	10 min: Guided meditation<br>30 min: The impact of stress on our well-being<br>10 min: Exercise 1. Recognizing our signs of stress<br>10 min: Exercise 2. Hatha yoga<br>10 min: Break<br>20 min: Exercise 3. Body sweeping<br>30 min: Group sharing of effects, questioning and deepening of the material taught (group discussion for qualitative collection)<br>10 min: Guided meditation
Session 7	10 min: Guided meditation<br>30 min: Integrating mindfulness into our lives<br>10 min: Exercise 1: Sitting meditation<br>10 min: Exercise 2. Hatha yoga<br>10 min: Break<br>20 min: Integrating mindfulness into our lives (continued)<br>30 min: Group sharing of effects, questioning and deepening of the material taught (group discussion for qualitative collection)<br>10 min: Guided meditation
Session 8	10 min: Guided meditation<br>30 min: Conclusion: looking to the future<br>10 min: Exercise 1: What I've learned<br>10 min: Exercise 2. Meditation in motion<br>10 min: Break<br>20 min: Conclusion: looking to the future (continued)<br>30 min: Group sharing of effects, questioning and deepening of the material taught (group discussion for qualitative collection)<br>10 min: Guided meditation
