# Supplementary material for: Feasibility of a Virtual Mindfulness-Based Stress Reduction Intervention for Individuals With Chronic Pain: Pilot Randomized Controlled Trial
Source: JMIR Form Res. 2026 Sep 23;10:e98632. doi: 10.2196/98632 (PMC13600662; doi:10.2196/98632)
Supplement: Supplementary file 2 [file formative-v10-e98632-s002.pdf]

Table. Descriptive statistics and linear mixed model results as a sensitivity analysis primary analysis of clinical outcome measures among participants with complete data at all three assessment points (n=37; MBSR=19, control=18), assessed at baseline (T1), post-intervention (T2), and three-month follow-up (T3), among adults with chronic pain randomized to an eight-week virtual mindfulness-based stress reduction (MBSR) intervention or a no-intervention control condition in a pilot randomized controlled trial conducted in Quebec, Canada (May 2021 to February 2022).

Outcome	**MCID (study citation)**	**Experimental (n= 19)**			**Control (n= n= 18)**			**Linear Mixed Model**		
		**T1**	**T2**	**T3**	**T1**	**T2**	**T3**	**Time**	**Group**	**Time*group**
				**Pain Disability Index**						
Overall	8.5 to 9.5 [65]	36.2 ± 2.3	31.7 ± 2.9	24.1 ± 2.9 † ‡ ¤ *	37.6 ± 2.4	37.0 ± 2.9	34.0 ± 3.0 ¤	**<0.001**	0.115	**0.040**
				**Pain Catastrophizing Scale**						
Overall	8 to 11 [66]	26.6 ± 2.2	18.1 ± 2.5 *	17.1 ± 2.4 *	28.4 ± 2.3	25.0 ± 2.5	22.8 ± 2.4	**<0.001**	0.141	0.061
				**Chronic Pain Acceptance Questionnaire**						
Overall	NA	28.7 ± 1.3	27.7 ± 1.3	28.6 ± 1.3	29.5 ± 1.3	29.5 ± 1.3	31.3 ± 1.3	0.167	0.301	0.511
				**Pain Self-Efficacy Questionnaire**						
Overall	5.5 to 8.5 [67]	36.0 ± 2.4	41.2 ± 1.8	40 ± 2.3	32.0 ± 2.4	33.2 ± 1.9	35.7 ± 2.4	0.116	**0.043**	0.219
				**PROMIS-10 Global Health**						
T-Score Physical health	2 to 6 [68]	36.3 ± 0.9	40.9 ± 1.1 ¥ # *	38.1 ± 1.1 †	34.9 ± 0.9	35.0 ± 1.6 ¥	35.6 ± 1.1	**0.004**	**0.012**	**0.004**
T-Score Mental health		41.2 ± 1.7	45.0 ± 1.5 *	44.8 ± 1.5 *	40.8 ± 1.8	41.1 ± 1.5	41.3 ± 1.5	0.070	0.217	0.165
Global health		2.5 ± 0.1	2.8 ± 0.1 #	2.8 ± 0.1	2.6 ± 0.1	2.4 ± 0.1	2.6 ± 0.1	0.403	0.469	**0.012**
Social activities & role		2.6 ± 0.1	2.8 ± 0.2	2.9 ± 0.1	2.1 ± 0.2	2.5 ± 0.2	2.5 ± 0.1	0.056	0.094	0.741
Pain intensity (0-10)		6.7 ± 0.2	5.6 ± 0.3	5.6 ± 0.3	6.7 ± 0.3	6.5 ± 0.3	5.9 ± 0.3	**0.006**	0.361	0.184
				**PROMIS-29 T-Score**						
Pain interference	2 to 6 [68]	64.2 ± 1.1	59.7 ± 1.0 *	60.1 ± 1.3 *	66.7 ± 1.1	64.0 ± 1.0 *	64.8 ± 1.4	**<0.001**	**0.012**	0.288
Participation in social activities		42.2 ± 1.5	47.7 ± 1.6 # ¥ *	43.4 ± 1.7 †	37.8 ± 1.6	40.0 ± 1.6 ¥ *	41.7 ± 1.8 ‡ *	**0.004**	**0.032**	**0.028**

Physical function	39.4 ± 1.2	40.0 ± 1.1	40.4 ± 1.4	35.4 ± 1.2	36.5 ± 1.1	35.8 ± 1.5	0.152	**0.026**	0.751
Fatigue	60.6 ± 1.6	56.5 ± 1.9 *	55.9 ± 1.9 *	61.5 ± 1.7	61.0 ± 1.9	59.4 ± 2.0 *	0.059	0.166	0.424
Anxiety/fear	61.5 ± 1.8	54.6 ± 1.8 *	55.4 ± 2.0 *	61.5 ± 1.9	59.9 ± 1.9	57.8 ± 2.0 *	**<0.001**	0.302	0.095
Sleep disturbance	59.1 ± 1.6	53.8 ± 1.8 *	53.5 ± 2.0 *	60.6 ± 1.6	57.9 ± 1.9 *	56.1 ± 2.1 *	**0.014**	0.198	0.606
Depression/sadness	58.1 ± 1.5	54.8 ± 1.8 *	53.3 ± 1.9 *	59.4 ± 1.6	59.9 ± 1.9	57.5 ± 1.9	**0.009**	0.120	0.288

MCID: Minimally Clinical Important difference, post-hoc within-group differences with Bonferroni corrections within (T1-T2: #, T2-T3: †, T1-T3: ‡), and between-group (at T1: ˆ, T2: **¥**, T3: ¤), Denotes minimum clinically important difference from baseline values: ***,** NA: Not available
