# Supplementary material for: Feasibility of a Virtual Mindfulness-Based Stress Reduction Intervention for Individuals With Chronic Pain: Pilot Randomized Controlled Trial
Source: JMIR Form Res. 2026 Sep 23;10:e98632. doi: 10.2196/98632 (PMC13600662; doi:10.2196/98632)
Supplement: Supplementary file 3 [file formative-v10-e98632-s003.pdf]

Table. Post-hoc descriptive analysis of outcome measures by intervention engagement tier at baseline (T1), post-intervention at 8 weeks (T2), and 3-month follow-up (T3) among MBSR participants (n=30) in a pilot randomized controlled trial of virtual mindfulness-based stress reduction for chronic pain in Quebec, Canada. Data are presented as mean ± standard deviation

Group	**MCID (study citation)**	T1	T2	T3
**Pain Disability Index**				
≥4 sessions, adequate practice	8.5 to 9.5 [65]	38.3 ± 7.4, n= 19	33.4 ± 12.0, n= 17	28.0 ± 12.3*, n= 17
≥4 sessions, inadequate practice		35.0 ± 19.0, n= 3	27.0 ± 28.3*, n= 2	12.5 ± 9.2*, n= 2
≤3 sessions, inadequate practice		30.7 ± 9.4, n= 8	22.0*, n= 1	0.0*, n= 1
**Pain Catastrophizing Scale**				
≥4 sessions, adequate practice	8 to 11 [66]	28.7 ± 9.4, n= 19	20.7 ± 10.9*, n= 17	19.0 ± 10.6*, n= 17
≥4 sessions, inadequate practice		17.3 ± 7.6, n= 3	9.0 ± 2.8*, n= 2	14.0 ± 7.1, n= 2
≤3 sessions, inadequate practice		25.2 ± 12.6, n= 8	8.0*, n= 1	10.0*, n= 1
**Chronic Pain Acceptance Questionnaire**				
≥4 sessions, adequate practice	NA	28.6 ± 6.7, n= 19	28.0± 6.9, n= 17	28.2 ± 6.1, n= 17
≥4 sessions, inadequate practice		28.3 ± 7.6, n= 3	24.5 ± 13.4, n= 2	29.0 ± 9.9, n= 2
≤3 sessions, inadequate practice		30.2 ± 4.5, n= 8	30.0, n= 1	35.0, n= 1
**Pain Self-Efficacy Questionnaire**				
≥4 sessions, adequate practice	5.5 to 8.5 [67]	34.4 ± 9.8, n= 19	39.9 ± 7.6*, n= 17	38.6 ± 8.5*, n= 17
≥4 sessions, inadequate practice		40.0 ± 6.0, n= 3	46.5 ± 0.7*, n= 2	45.5 ± 7.8*, n= 2
≤3 sessions, inadequate practice		37.6 ± 11.2, n= 8	45.0*, n= 1	45.0*, n= 1
**PROMIS-10 Global Health**				
T-Score Physical health				
≥4 sessions, adequate practice	2 to 6 [68]	35.8 ± 3.4, n= 19	40.1 ± 5.1*, n= 17	36.8 ± 2.7, n= 16

≥4 sessions, inadequate practice		36.1 ± 10.6, n= 3	44.9 ± 5.6*, n= 2	43.8 ± 2.4*, n= 2
≤3 sessions, inadequate practice		34.4 ± 6.2, n= 8	47.5*, n= 1	48.4*, n= 1
T-Score Mental health				
≥4 sessions, adequate practice	2 to 6 [68]	39.7 ± 7.6, n= 19	44.2 ± 6.7*, n= 17	44.0 ± 6.7*, n= 16
≥4 sessions, inadequate practice		51.5 ± 7.5, n= 3	50.5 ± 3.6, n= 2	49.4 ± 2.0*, n= 2
≤3 sessions, inadequate practice		40.7 ± 4.8, n= 8	46.0*, n= 1	48.2*, n= 1
Global health				
≥4 sessions, adequate practice	2 to 6 [68]	2.5 ± 0.7, n= 19	2.7 ± 0.5, n= 17	2.7 ± 0.6, n= 16
≥4 sessions, inadequate practice		3.0 ± 0, n= 3	3.5 ± 0.7, n= 2	3.5 ± 0.7, n= 2
≤3 sessions, inadequate practice		2.8 ± 1.1, n= 8	3.0, n= 1	3.0, n= 1
Social activities & role				
≥4 sessions, adequate practice	2 to 6 [68]	2.6 ± 0.9, n= 19	2.8 ± 1.0, n= 17	2.8 ± 0.7, n= 16
≥4 sessions, inadequate practice		3.6 ± 1.1, n= 3	3.0 ± 0, n= 2	3.5 ± 0.7, n= 2
≤3 sessions, inadequate practice		2.6 ± 0.9, n= 8	3.0, n= 1	3.0, n= 1
Pain intensity (0 to 10)				
≥4 sessions, adequate practice	2 to 6 [68]	6.7 ± 1.5, n= 19	5.5 ± 1.7, n= 17	5.7 ± 1.4, n= 16
≥4 sessions, inadequate practice		7.0 ± 1.7, n= 3	6.5 ± 0.7, n= 2	5.5 ± 0.7, n= 2
≤3 sessions, inadequate practice		7.2 ± 1.5, n= 8	7.0, n= 1	5.0*, n= 1
**PROMIS-29**				
Participation in social activities				
≥4 sessions, adequate practice	2 to 6 [68]	39.1 ± 6.3, n= 19	46.2 ± 6.6*, n= 17	41.2 ± 5.8*, n= 16
≥4 sessions, inadequate practice		51.5 ± 7.1, n= 3	55.2 ± 12.6*, n= 2	56.0 ± 3.4*, n= 2

≤3 sessions, inadequate practice		38.7 ± 10.3, n= 8	50.2*, n= 1	53.6*, n= 1
Anxiety/fear				
≥4 sessions, adequate practice	2 to 6 [68]	62.6 ± 6.9, n= 19	53.7 ± 9.7*, n= 17	55.6 ± 9.1*, n= 16
≥4 sessions, inadequate practice		55.7 ± 5.2, n= 3	58.7 ± 4.1, n= 2	52.9 ± 1.6*, n= 2
≤3 sessions, inadequate practice		62.0 ± 6.9, n= 8	57.5*, n= 1	57.5*, n= 1
Depression/sadness				
≥4 sessions, adequate practice	2 to 6 [68]	59.5 ± 7.2, n= 19	54.3 ± 9.7*, n= 17	52.8 ± 9.1*, n= 16
≥4 sessions, inadequate practice		54.2 ± 4.4, n= 3	56.5 ± 3.3, n= 2	55.1 ± 1.4, n= 2
≤3 sessions, inadequate practice		60.2 ± 8.6, n= 8	57.5*, n= 1	58.9, n= 1
Fatigue				
≥4 sessions, adequate practice	2 to 6 [68]	63.1 ± 7.7, n= 19	57.8 ± 9.1*, n= 17	57.3 ± 9.5*, n= 16
≥4 sessions, inadequate practice		58.4 ± 8.6, n= 3	54.2 ± 1.4*, n= 2	51.9 ± 4.6*, n= 2
≤3 sessions, inadequate practice		60.4 ± 7.9, n= 8	48.2*, n= 1	43.1*, n= 1
Pain interference				
≥4 sessions, adequate practice	2 to 6 [68]	65.4 ± 5.3, n= 19	60.2 ± 5.0*, n= 17	61.1 ± 5.0*, n= 16
≥4 sessions, inadequate practice		58.9 ± 8.3, n= 3	56.2 ± 8.5*, n= 2	52.8 ± 1.9*, n= 2
≤3 sessions, inadequate practice		64.0 ± 5.6, n= 8	55.9*, n= 1	58.5*, n= 1
Physical function				
≥4 sessions, adequate practice	2 to 6 [68]	37.6 ± 4.3, n= 19	38.5 ± 3.9*, n= 17	38.7 ± 6.1*, n= 16
≥4 sessions, inadequate practice		43.4 ± 12.5, n= 3	48.3 ± 12.3*, n= 2	50.4 ± 9.3*, n= 2
≤3 sessions, inadequate practice		40.2 ± 8.2, n= 8	47.8*, n= 1	47.8*, n= 1
Sleep disturbance				

≥4 sessions, adequate practice	2 to 6 [68]	59.6 ± 6.4, n= 19	54.9 ± 7.8*, n= 17	54.1 ± 9.1*, n= 16
≥4 sessions, inadequate practice		53.7 ± 1.3, n= 3	46.4 ± 7.4*, n= 2	52.2 ± 1.1*, n= 2
≤3 sessions, inadequate practice		55.0 ± 10.5, n= 8	49.6*, n= 1	45.8*, n= 1
**PGICS**				
≥4 sessions, adequate practice	1.35 [69]	-	3.0 ± 1.5*, n= 17	-
≥4 sessions, inadequate practice		-	2.0 ± 0.0*, n= 2	-
≤3 sessions, inadequate practice		-	3.0*, n= 1	-

CPAQ: Chronic Pain Acceptance Questionnaire, MCID: Minimal Clinically Important Difference, NA: Not Applicable, PCS: Pain Catastrophizing Scale, PDI: Pain Disability Index, PGICS: Patient Global Impression of Change Scale, PROMIS-10: Patient-Reported Outcomes Measurement Information System-10, PROMIS-29: Patient-Reported Outcomes Measurement Information System-29, PSEQ: Pain Self-Efficacy Questionnaire, *Denotes a change from baseline meeting or exceeding the minimum clinically important difference
