# Supplementary material for: Feasibility of a Virtual Mindfulness-Based Stress Reduction Intervention for Individuals With Chronic Pain: Pilot Randomized Controlled Trial
Source: JMIR Form Res. 2026 Sep 23;10:e98632. doi: 10.2196/98632 (PMC13600662; doi:10.2196/98632)
Supplement: Supplementary file 4 [file formative-v10-e98632-s004.pdf]

**Feasibility of a Virtual Mindfulness-Based Stress Reduction Intervention for Individuals with Chronic Pain: A Double-Blind Pilot Randomized Controlled Trial**

Supplementary file

**Table 1.** CONSORT checklist

| | | Reporting Item | Page Number |
|---|---|---|---|
| **Title and Abstract** | | | |
| Title | #1a | Identification as a randomized trial in the title. | 1 |
| Abstract | #1b | Structured summary of trial design, methods, results, and conclusions | 1 |
| **Introduction** | | | |
| Background and objectives | #2a | Scientific background and explanation of rationale | 2-3 |
| Background and objectives | #2b | Specific objectives or hypothesis | 3 |
| **Methods** | | | |
| Trial design | #3a | Description of trial design (such as parallel, factorial) including allocation ratio. | 3-5 |
| Trial design | #3b | Important changes to methods after trial commencement (such as eligibility criteria), with reasons | n/a |
| Participants | #4a | Eligibility criteria for participants | 4 |
| Participants | #4b | Settings and locations where the data were collected | 4 |
| Interventions | #5 | The experimental and control interventions for each group with sufficient details to allow replication, including how and when they were actually administered | 4-5 |
| Outcomes | #6a | Completely defined prespecified primary and secondary outcome measures, including how and when they were assessed | 5-7 |
| Sample size | #7a | How sample size was determined. | 8-9 |
| Sample size | #7b | When applicable, explanation of any interim analyses and stopping guidelines | n/a |

| | | | |
|---|---|---|---|
| Randomization - Sequence generation | #8a | Method used to generate the random allocation sequence. | 5 |
| Randomization - Sequence generation | #8b | Type of randomization; details of any restriction (such as blocking and block size) | 5 |
| Randomization - Allocation concealment mechanism | #9 | Mechanism used to implement the random allocation sequence (such as sequentially numbered containers), describing any steps taken to conceal the sequence until interventions were assigned | 5 |
| Randomization - Implementation | #10 | Who generated the allocation sequence, who enrolled participants, and who assigned participants to interventions | 5 |
| Blinding | #11a | If done, who was blinded after assignment to interventions (for example, participants, care providers, those assessing outcomes) and how. | 5 |
| Blinding | #11b | If relevant, description of the similarity of interventions | n/a |
| Statistical methods | #12a | Statistical methods used to compare groups for primary and secondary outcomes | 8-9 |
| Statistical methods | #12b | Methods for additional analyses, such as subgroup analyses and adjusted analyses | 8-9 |
| Outcomes | #6b | Any changes to trial outcomes after the trial commenced, with reasons | n/a |
| **Results** | | | |
| Participant flow diagram (strongly recommended) | #13a | For each group, the numbers of participants who were randomly assigned, received intended treatment, and were analysed for the primary outcome | 9 |
| Participant flow | #13b | For each group, losses and exclusions after randomization, together with reason | 9-10 |
| Recruitment | #14a | Dates defining the periods of recruitment and follow-up | 9-10 |
| Recruitment | #14b | Why the trial ended or was stopped | n/a |
| Baseline data | #15 | A table showing baseline demographic and clinical characteristics for each group | 10-11 |

| | | | |
|---|---|---|---|
| Numbers analysed | #16 | For each group, number of participants (denominator) included in each analysis and whether the analysis was by original assigned groups | 10-11 |
| Outcomes and estimation | #17a | For each primary and secondary outcome, results for each group, and the estimated effect size and its precision (such as 95% confidence interval) | 11-19 |
| Outcomes and estimation | #17b | For binary outcomes, presentation of both absolute and relative effect sizes is recommended | n/a |
| Ancillary analyses | #18 | Results of any other analyses performed, including subgroup analyses and adjusted analyses, distinguishing pre-specified from exploratory | n/a |
| Harms | #19 | All important harms or unintended effects in each group (For specific guidance see CONSORT for harms) | 18-19 |
| **Discussion** | | | |
| Limitations | #20 | Trial limitations, addressing sources of potential bias, imprecision, and, if relevant, multiplicity of analyses | 23 |
| Interpretation | #22 | Interpretation consistent with results, balancing benefits and harms, and considering other relevant evidence | 19-23 |
| Registration | #23 | Registration number and name of trial registry | 4 |
| Generalisability | #21 | Generalisability (external validity, applicability) of the trial findings | 19-23 |
| **Other information** | | | |
| Interpretation | #22 | Interpretation consistent with results, balancing benefits and harms, and considering other relevant evidence | 19-23 |
| Registration | #23 | Registration number and name of trial registry | 4 |
| Protocol | #24 | Where the full trial protocol can be accessed, if available | n/a |
| Funding | #25 | Sources of funding and other support (such as supply of drugs), role of funders | 24 |
